# Brain readiness and the nature of language

**DOI:** 10.3389/fpsyg.2015.01376

**Published:** 2015-09-09

**Authors:** Denis Bouchard

**Affiliations:** Département de linguistique, Université du Québec à MontréalMontreal, QC, Canada

**Keywords:** language evolution, evolvability, linguistic signs, brain readiness, self-organization

## Abstract

To identify the neural components that make a brain ready for language, it is important to have well defined linguistic phenotypes, to know precisely what language is. There are two central features to language: the capacity to form signs (words), and the capacity to combine them into complex structures. We must determine how the human brain enables these capacities. A sign is a link between a perceptual form and a conceptual meaning. Acoustic elements and content elements, are already brain-internal in non-human animals, but as categorical systems linked with brain-external elements. Being indexically tied to objects of the world, they cannot freely link to form signs. A crucial property of a language-ready brain is the capacity to process perceptual forms and contents offline, detached from any brain-external phenomena, so their “representations” may be linked into signs. These brain systems appear to have pleiotropic effects on a variety of phenotypic traits and not to be specifically designed for language. Syntax combines signs, so the combination of two signs operates simultaneously on their meaning and form. The operation combining the meanings long antedates its function in language: the primitive mode of predication operative in representing some information about an object. The combination of the forms is enabled by the capacity of the brain to segment vocal and visual information into discrete elements. Discrete temporal units have order and juxtaposition, and vocal units have intonation, length, and stress. These are primitive combinatorial processes. So the prior properties of the physical and conceptual elements of the sign introduce combinatoriality into the linguistic system, and from these primitive combinatorial systems derive concatenation in phonology and combination in morphosyntax. Given the nature of language, a key feature to our understanding of the language-ready brain is to be found in the mechanisms in human brains that enable the unique means of representation that allow perceptual forms and contents to be linked into signs.

## Introduction

The main point of this paper is that the central trait of human language is the capacity to form signs by linking perceptual forms and meanings^1^. This predicts that the core mechanisms that make a brain ready for language are those that enable this capacity. Moreover, switching from a computational view of language to a sign-based theory provides a unified approach to the^1^ functioning of the main subsystems of language. The perceptual and conceptual substances of signs create a system that reaches a level of such complexity that it triggers self-organization, deriving specific properties of signs, as well as the basic structuring of language in its phonology, semantics, and syntax.

## The core competence for language

A language-ready brain raises two evolutionary puzzles: a puzzle of emergence and a puzzle of design (Hoefler, 2009, p. 1). The puzzle of emergence addresses the problem of bridging the gap from a stage where our ancestors had no language to a stage where they had language as we know it today. How and why did language emerge in humans and not in other species?

Lewontin (1998) raises strong doubts about the possibility of reconstructing the evolutionary history and the causal mechanisms of the acquisition of linguistic competence (and cognition in general). He emphasizes the near impossibility to come up with evidence “that there was heritable variation for, say, linguistic ability, in our remote ancestors when the human species was still evolving into its present form and that those who possessed this ability, in the remote past, left more offspring by virtue of that ability” (p. 111). So it is extremely difficult for the standard theory of evolution by natural selection to inform us on how language, and more generally, cognition arose and spread and changed. As he points out, humans had an ancestor in common with the chimpanzee and the gorilla about 10 million years ago. So 20 million years of evolution separate us from our closest relatives. During that period, “a major difference in the consequences of cognitive power has taken place during human evolution that makes the cognitive difference between gorillas and chimpanzees trivial compared to our cognitive distance from them” (p. 116). Evolved forms may diverge very dramatically in a relatively short period of time. Lewontin gives the example of cows, goats, and deer that differentiated 10 million years ago. Therefore, it is unlikely that we can determine—even approximately—when our linguistic capacity emerged in our ancestry. In addition, a trait may derive from analogy just as well as from homology. Moreover, we cannot measure the actual reproductive advantages of cognition or language. Fossils, furthermore, are of very little help concerning cognition, and often we cannot even be sure whether a fossil is from an ancestor or some relative on another branch of the bush-like relations between species. So we cannot tell what our immediate non-linguistic ancestors were like cognitively. Almost two decades after the publication of his paper, the problem still appears to be substantial, though advances in our knowledge of genes open some research avenues concerning heritable variation, even for remote ancestors.

Nevertheless, there is room for testable theories about what language is, what brain mechanisms this requires, and whether some of these brain mechanisms are unique to humans at least compared to other current species. As we progress in our understanding of the human brain, we can compare it with the neuro-anatomy of related species and see how they differ in form and function. We can pinpoint some current neurological distinctive trait(s) that enable(s) language, and hence determine WHAT made language emerge. Regarding WHEN and HOW the organism evolved to get that change, we can only speculate. But at least we can elaborate a theory that passes the test of evolvability: if a theory can show how some actual neuro-anatomical element enables language as we know it, then that theory is in accord with the fact that an organism with a language-ready brain is an evolvable organism, because this neuro-anatomical element can indeed develop according to the laws and principles of biological evolution, since it exists in human brains. Moreover, the nature of the neuro-anatomical trait can give us an indication of what it could have come from. This is particularly the case if language is a side effect of the neuro-anatomical trait, as I argue below: the other functional effects of the trait can further restrict the possible scenarios.

This brings us to the second evolutionary puzzle, the question of design: how and why did language evolve with the properties that we observe rather than some other set? To identify the components that make a brain ready for language, neuroscientists must know precisely what such a brain must do, hence ultimately, what language is. Not that the brain mechanisms will somehow be analogical to the functional aspects of language: examples abound where it has been shown that the neural substrates or the mechanisms supporting behavior, are not predicted by psychological models. However, we must understand precisely what language is and have well-defined linguistic phenotypes to search for the neural substrates that enable these phenotypes.

There are numerous properties that have been attributed to language. Many have been recently proposed and many are not widely accepted because they depend on narrow theoretical assumptions. It would be a formidable task to look at hundreds of properties in exploring the language-readiness of the brain, and probably futile in many instances since the properties are probably ephemeral. It is more productive to investigate two properties of language for which there is a long-standing and broad consensus among scholars—the capacity to form signs (words, morphemes), and the capacity to combine them into complex structures:

“at least two basic problems arise when we consider the origins of the faculty of language […]: first, the core semantics of minimal meaning-bearing elements, including the simplest of them; and second, the principles that allow infinite combinations of symbols, hierarchically organized, which provide the means for use of language in its many aspects” (Chomsky, 2005, p. 4).

If we can explain how the brain is ready for these two basic properties, how it enables them, we are heading in the right direction. However, if we consider what the founder of the most prominent theoretical model in linguistics says about the evolution of these two properties, the prospects look rather dim. Concerning the capacity to form signs, Chomsky (2010) says that it is “of totally mysterious origin.” Moreover, though he has contributed to a very influential paper on the origin of linguistic combinatoriality (Hauser et al., 2002), Chomsky and some of his colleagues now believe that the origin of combinatoriality is also a mystery, as indicated in the very title of their paper: “The mystery of language evolution” (Hauser et al., 2014).

The problem is further amplified by the fact that, despite recent attempts to limit it, the current model still relies on a large set of innate, language-specific conditions—Universal Grammar (UG)—which is a repertory of unexplained properties (Chomsky, 2007, p. 19)^2^. UG is therefore a highly problematic component from an explanatory point of view, since the richer the set of language-specific brain features, the harder it will be to account for it: “Aspects of the computational system that do not yield to principled explanation fall under UG, to be explained **somehow in other terms** [my emphasis, DB], questions that may lie beyond the reach of contemporary inquiry, Lewontin (1998) has argued” (Chomsky, 2007, p. 24). This is as close as one can get to saying that UG is also an unsolved mystery, maybe even an unsolvable one^3^.

The three mysteries are not simply subcases of the difficulty to reconstruct evolutionary history and the causal mechanisms of the acquisition of linguistic competence: they are also problems of evolvability. The UG model appears incapable of providing a principled explanation based on some neuro-anatomical elements that would account for the numerous language-specific components it postulates. Brain readiness and evolvability are closely linked, so evolvability is an important test for linguistic theories: the traits that a linguistic theory requires of the human brain must be highly plausible according to the known laws and principles of biological evolution. We may not be able to trace the evolutionary path of how language emerged, but we can evaluate the degree of evolvability of a linguistic model, its plausibility given known laws of evolution.

In the face of the triple mystery assessment, we might judge that the evolvability of the language-ready brain is too hard a problem and decide to simply drop it. But scientists don't like to give up. If the problem appears insurmountable from the perspective a theory, however widely scholars adhere to it, its apparent incapacity to deal with such core issues as signs, combinatoriality and language-specific conditions in general, can be a motive to scrutinize that theory to figure out why it fails in this respect, and to use this assessment to elaborate an alternative model that can adequately address the core issues. Proponents of UG, and those who share the mystery assessment about language such as Lewontin (1998), all put a high emphasis on the property of discrete infinity found in language, which is assumed to be the core property of the language phenotype: “the core competence for language is a biological capacity shared by all humans and distinguished by the central feature of discrete infinity—the capacity for unbounded composition of various linguistic objects into complex structures” (Hauser et al., 2014, p. 2). This is understandable from a historical background. Generative grammar was born in the context of emerging tools in mathematical logic. For the first time, these tools provided the means to formalize recursion, which had been informally recognized as a property of language for some time (cf. Humboldt's infinite use of finite means). In this context, the most striking characteristic of human language is its discrete infinity. It is tempting to see discrete infinity as an essential property of language, and to put the corresponding technical tools of recursion at the heart of the model. It is then natural to assume that recursion is the crucial distinctive property of human language.

But this core assumption leads to a triple mystery. We should therefore question that assumption. The language phenotype, like all “facts,” is a set of observational propositions which are part of the theory: they are not external to the theory and independent (Lakatos, 1970), and their status can be questioned like any other proposition, particularly in the face of an overwhelming problem such as when a theory leads to a shroud of mysteries. It turns out that the assumption of the centrality of recursion and discrete infinity, though shared by many language scientists, is incorrect. Although it is an observable trait of language, it is not the core phenotype it is assumed to be, but a side effect. The core competence for language is the capacity to take elements from two substances with no logical or natural connection between their elements—perceptual forms and meanings—and to link them into signs (words, morphemes). This capacity to form Saussurean signs is the sole distinctive trait of human language. The fact that only human language has discrete infinity does not imply that recursion is a distinguishing mechanism. This mechanism is uniquely human; however, it is not original: it actually arises from prior elements of the two substances of signs that contain primitive combinatorial processes and produce the effects of recursion^4^.

To see this, let us now turn to the detailed properties of linguistic signs.

## The sign theory of language

A linguistic sign is generally presented as involving two elements—a meaning and a form—and a link between the two. Saussure (1916) introduced the terms signified and signifier to emphasize that this linking is purely mental, established by speakers. I use the terms “concept” and “percept” in this spirit: they are dynamical mental creations, cognitive structures (see Jackendoff, 2002, ch. 10). This is an oversimplification, however. A linguistic form (signifier/percept) is a mental state linked to an acoustic/visual material element: this element is not linguistic but in the domain of the sciences that deal with the physical and mental properties of acoustic perception and production (Henceforth, I will only discuss acoustic material of the oral modality, but the ideas carry over to the gestural modality). Similarly, a linguistic meaning (signified/concept) is a mental state linked to a psychological element, a chunk of cognition that the mental state evokes: this element also is not linguistic but in the domain of the sciences that deal with psychological phenomena related to thought. It is only when a language establishes Link 1 between a representation of a perceptual element and a representation of a conceptual element that these are linguistically relevant and become a signifier and a signified.

**(1)** Figure 1

**Figure 1:**
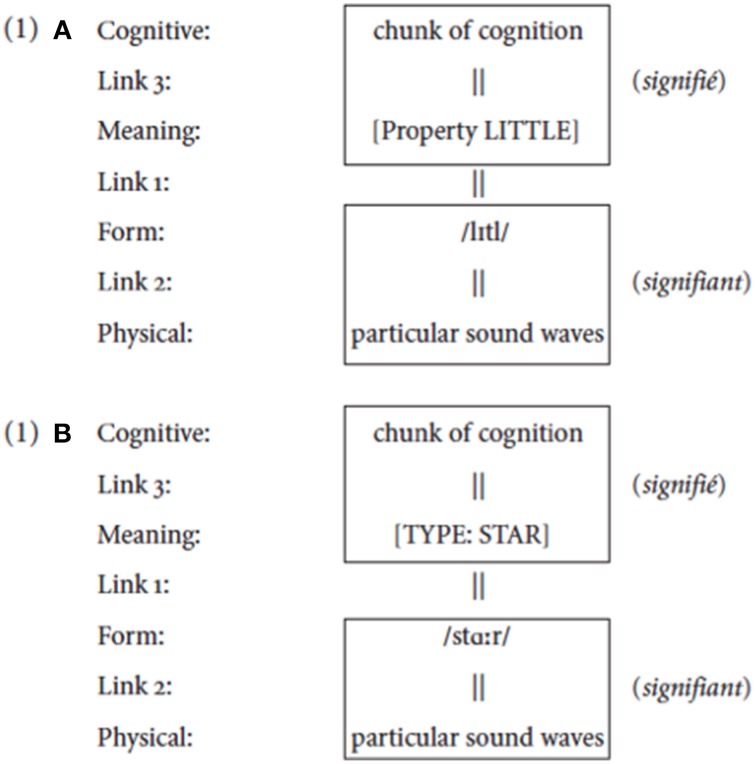
**The structure of a linguistic sign**. **(A)** shows the structure of the word “little.” Its linguistic elements are its meaning (here simply represented as LITTLE), which is related to the combination of phonemes that are its form. These linguistic elements are each related to elements outside the realm of language: a certain chunk of cognition for the meaning LITTLE, and physical sound waves for its form. **(B)** shows the structure corresponding to the word “star.”

The linguistically crucial part of a sign is a reciprocal predication: it is the systematic attribution of a vocal form and a meaning to each other. The link between signifier and signified is not determined by logic or by intrinsic properties in the nature of the phonic-acoustic or conceptual substances: it is purely linguistic. The properties of the substances to which the signifiers and signifieds are linked cannot explain why a particular phonetic entity is tagged as the signifier of a certain meaning or why a particular conceptual entity is tagged as the signified of a certain form. These links are not due to natural causes, but rather are arbitrary because the nature of the sounds that our phonatory articulators produce and the nature of the concepts that our conceptual system constructs are so different that they cannot entertain a meaningful, logical, or iconic relation (Saussure, 1916, pp. 155–156).

Now consider syntax. If we look at it in terms as neutral as possible, syntax is minimally defined as the processes by which signs are combined. Consider a simple example of the syntactic combination of the two signs *little* and *star*. Each sign is complex by definition—a form resulting from the union of a signified and a signifier. Syntax does not combine just signifiers or just signifieds, it combines relations between signifiers and signifieds, i.e., signs. Since signified and signifier are irreducibly united, any operation applying to one is reflected on the other. So when two signs are combined by a relation R, R operates simultaneously on both their signifieds and their signifiers, as shown in the combination of *little* and *star* in (2).

**(2)** Figure 2

**Figure 2:**
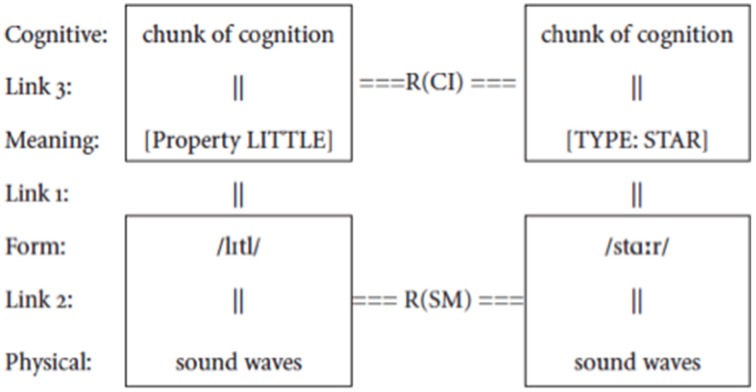
**The structure of a combinatorial sign**. A syntactic combination of words such as *little* and *star* is realized by a combinatorial sign which operates simultaneously on their meanings, creating a relation R(CI) at the conceptual-intentional level, and on their forms, creating a relation R(SM) at the sensory-motor level.

Since R operates simultaneously on both the signifieds and the signifiers of the signs in (2), it is itself a sign. I will refer to this set of signs that combine syntactic elements as combinatorial signs (C-signs), to distinguish them from the more familiar unit signs (U-signs), namely words/morphemes. This immediately raises two questions: What is the signifier of a C-sign? What is the signified of a C-sign? As already indicated in Bouchard (1996, 2002), the signifier of a C-sign will take whatever form a language arbitrarily selects from among those that our physiology provides as a combinatorial percept in the modality of that language. These forms are drawn from physical traits of the forms of words. For instance, a first trait in an oral language is that vocal units appear linearly ordered. So signifiers made up of these vocal units can share a temporal edge—they can be temporally juxtaposed: two signifiers can be ordered next to one another, and this can be grammatically significant in the system of a language. For instance, in (3), it is grammatically significant that *saw* and *John* are juxtaposed, but not that *John* and *yesterday* are juxtaposed: the juxtaposition of *yesterday* is grammatically relevant only with respect to the phrase *saw John* (or *Mary saw John* under different assumptions).

(3) Mary saw John yesterday.

The order of juxtaposition is also frequently significant, as in the pairs in (4):

(4) a. John saw Mary—Mary saw John
b. John is sick—Is John sick?

A second trait is that the two signifiers can share a temporal space, as when a modulation is superimposed on the phonemes of a constituent: one signifier is the intonation placed on the other signifier, such as an intonation expressing a question (4b). Other possible superimposed elements are stress and length^5^. All these combinatorial percepts depend on the physiological traits of the modality, so they vary across modalities. For instance, the visual–gestural channel of sign languages has more types of combinatorial percepts because it uses more articulators and more dimensions than the auditory–oral channel (Bouchard, 1996).

The set of possible signifiers for a C-sign is extremely restricted because the set of physiological relational vocal percepts is small. So arbitrariness is limited by what are ultimately principles of physical science, as Thompson (1917) anticipated for biological systems in general. Languages vary in their choices of signifiers among these combinatorial percepts, as expected in the light of arbitrariness. For instance, the syntactic relation “direct object” can be expressed by any of these combinatorial signifiers: juxtaposition in the order V-NP or NP-V, a Case affix or a Case tone on the complement, an object affix or an object tone on the verb. This follows from Saussure's general principle of arbitrariness. There is no “reason of nature” for a language—let alone all languages—to choose any particular combinatorial signifier among those enabled by our physiology: any signifier is a possible candidate, because each one can optimally satisfy the requirement to encode meanings in a form. Indeed, each possibility is instantiated in some language or other. Languages choose from among the various possibilities of combinatorial signs, just as they arbitrarily choose from among the various possibilities of unit signs. Which combinatorial percepts are possible signifiers is not stipulated in some universal list, but is determined by prior properties of the perceptual substance of the modality of the particular language. Under this view, if there was no variation in the way languages express a relation such as “direct object,” if they all had the same signifier for it, this would be a most improbable accident, just as it would be if the signifier of a unit sign happened to be the same in all languages. Since Saussurean arbitrariness extends to C-signs, variation in syntax is a virtual necessity. Consequently, which particular combinatorial signifier is used in any specific case in a language must be learned just as much as any signifier at the word level. The numerous instances in which each language must choose a C-sign create the impression that languages can be amazingly different. But this is just an impression due to the cumulative effect of the choices; in fact, each choice of C-sign involves only one of the very few percepts that human physiology allows as the signifier of a C-sign. Though each combination is very simple, these combinatorial means cumulatively allow syntax to create organized groups of signs which can attain a very high degree of complexity overall.

Consider now the nature of the meaning of a C-sign, that is, the relation R at the conceptual-intentional level. The signified of R is a relation of predication. Predication, namely the capacity to attribute properties/information to objects, is a universal trait of human cognition. As Hurford (2007a, p. 527) indicates, “In the very earliest mental processes, long antedating language, binary structure can be found, with components that one can associate with the functions of identifying or locating an object and representing some information about it.”

In a combination of signs as in (2), the semantic part of the C-sign links two elements so that one adds its denotation as a restriction on the other, either in the usual sense for subject–predicate and topic–comment relations, as in (5), or in the sense of saturation, as in (6).

(5) a. John is sick/left early (the property of the VP is attributed to the subject).
b. that book, I really liked (the property of the comment is attributed to the topic).
(6) a. liked that book (the property of the direct object is attributed to the V, it saturates the verb).
b. in the kitchen (the property of the Noun Phrase is attributed to the locative preposition).

In summary, syntax is a set of combinatorial signs that allow the formation of complex signs. The perceptual form of a C-sign can only be either a juxtaposition or a superimposition of a vocal (or gestural) percept; this limitation on the combinatorial signifiers is due to properties of the human sensorimotor systems. The signified of a C-sign is predication, which was exapted from the pre-linguistic cognitive system of humans. Like other signs, combinatorial signs are subject to arbitrariness due to the nature of the two substances that they link. Therefore, which combinatorial signifier a language chooses for any particular predicative relation (i.e., “construction”) is arbitrarily selected from among those permitted by its modality. These are the main tenets of the Sign Theory of Language.

## The kind of brain mechanisms required for the formation of signs

A sign is a link between elements from domains of very different natures—a physical/perceptual form and a psychological/conceptual meaning. The core problem is to identify the brain mechanisms that enable links between these two kinds of elements. This neurological property (or set of properties) must be unique to the human brain since only humans have words: no other animal comes close to having equivalent signs detached from the immediate environment and as productively created. In Bouchard (2013), I suggest that these neuronal systems must have properties similar to the uniquely human systems of neurons discussed by Hurley (2008). These systems have the capacity to operate offline for input as well as output: they can be triggered not only by external events stimulating our perceptual systems but also by brain-internal events (including counterfactuals); they can also be activated while inhibiting output to any external (motoric) system. These Offline Brain Systems (OBS) are not specifically designed for language but they provide the crucial trait.

As early as 1891, Saussure understood that the fundamental duality of language is not in the linking of sound and meaning, but “resides in the duality of the vocal phenomenon AS SUCH, and of the vocal phenomenon AS A SIGN—of the physical fact (objective) and of the physical-mental fact (subjective)” (quoted in Bouquet and Engler, 2002, p. 20). The question is in what way, exclusive to humans, the vocal phenomenon enters into the mental domain, into the brain. Non-human animals can correctly classify and appropriately respond to stimuli, so acoustic elements, as well as informative content elements, are already brain-internal, but as categorical systems linked with brain-external elements. Being indexically tied to objects of the world, they are restricted in their mental activations and they cannot freely undergo linkings, they cannot form signs. Something different must be present in human brains. The brain mechanisms we are looking for must enable a vocal sound to be represented in the brain in a way detached from any brain-external phenomenon, as a purely brain-dependent entity, an activation of an OBS or something similar. Consequently, these representations of percepts can be arbitrarily linked to concepts: they can function as signifiers. I refer to these neural systems as Detached Representation systems (DR systems).

In addition to the physical element of a sign becoming a purely mental representation, the informational content of a sign is also different from that of an animal communication system unit. The content of an ACS unit is a category, i.e., a neural linking of similar results from sensory input, a class of input stimuli. This level is still linked to perceptual input—to the outside world. Even the signals that apes learn through intensive training remain at the level of action observation and embodied simulation of action triggered by external events. The content of a linguistic sign is at a more abstract level. It comes from human-specific cognemes that are abstracted from any sensory input or immediacy. This is the level at which detachment is attained. The concepts/meanings of signs do not represent or stand in for outer objects, but are brain activations that take internal events as inputs. This notion of “concept” is similar to the “amodal symbols” of Barsalou (1999) and the “types” of Penn et al. (2008):

“[…] only humans form general categories based on structural rather than perceptual criteria, find analogies between perceptually disparate relations, draw inferences based on the hierarchical or logical relation between relations, cognize the abstract functional role played by constituents in a relation as distinct from the constituents' perceptual characteristics, or postulate relations involving unobservable causes such as mental states and hypothetical physical forces. There is not simply a consistent absence of evidence for any of these higher-order relational operations in nonhuman animals; there is compelling evidence of an absence” (Penn et al., 2008, p. 110).

In order to be able to form linguistic signs, humans had to evolve brain systems that enable a more abstract representational level, so that concepts and percepts can be linked. It is not a percept *per se* that is linked with a concept *per se* in a linguistic sign, but a representation of the percept and a representation of the concept, i.e., a mental state corresponding to each of them, as we saw in figure (1). The crucial innovation is in the way some human neuronal systems function. Language did not emerge because there was environmental pressure for better communication or thought organization (though it brought leverage for both). It is not a system with a function of communication that emerged, nor with the function of organizing thought. It is a system of signs that emerged because elements from two very different substances met in the brain via their representations by new neuronal systems.

If the known laws of biology are extrapolated, we expect these brain systems to be in continuity with neuronal systems that are part of the machinery of the pre-linguistic brain, i.e., the brain of a prehuman species that has not yet achieved the capacity for detachment of the sort discussed above. Given biological continuity, it is likely that these are not radically different systems, but rather that they are offline activations of systems involving neurons in essentially the same parts of the brain.

In Bouchard (2013), I conjecture that these systems developed this novel kind of activation due to an increase in synaptic interactions that was triggered by several compounding factors. A large brain with a huge cortex offers a greatly increased potential for synaptic interactions. In addition, the more globular shape of the brain, with the thalamus in the middle, affords more cross-modular interactions (Boeckx, 2012). Moreover, alleles such as ApoE4 significantly improve synaptic repair; hence, they dramatically increase synaptic interactions. In addition, the long dependency during infancy feeds more cultural material into these additional brain capacities. With such a massive increase in synaptic interactions and complexity of circuitry due to biological changes and extensive cultural stimulation, a critical level was reached in hominid brains; some neuronal systems started being triggered by strictly internal brain events, introducing a new form of offline activation with no link to external events related to sensory inputs or motoric outputs. These strictly internal (offline) activations of some micro-anatomical structures represent a small evolutionary step: like the latching discussed by Russo and Treves (2011), they occur without altering the make-up of the neuronal network or any of its constituent properties. But DR systems have gigantic consequences: they enable brain activity of a novel kind and complexity, a unique representational capability that leads to higher level mentalizing.

The dramatic increases in both the number of neurons and the number connections between neuronal networks are instances where quantity produces quality, the brain activity becoming less input-driven and less rigid. It is not obvious that there is an immediate functional behavioral advantage for an individual to have this kind of detached brain activity. It can slow down reactions to the immediate environment, creating a sort of framing problem. From our current perspective, we see a quality in the innovation; but it may have come only in the long run—part of the pleiotropy of the innovation in brain activation that occurs due to material design, with no teleological push for an improvement of the individual's immediate well-being. Enhancements in the number of neurons and of connections lead to an increase in computational abilities and internal activity, but have little effect on the link between the brain and the perceptual systems interacting with the outer world. This kind of system does not evolve due to functional pressures: it takes on functions after its emergence. As Gould and Lewontin (1979) remark, a trait is not necessarily for something: it can just be a consequence.

This considerable upgrade in the quantity and quality of brain activity is like duplication in genes: other areas/systems can take over (Deacon, 2006), particularly given that the novel functional property of these micro-anatomical structures is less specialized, not tied to particular systems related to perception, but has a general representational capacity. Consequently, the various brain operations related to these systems are expected to exhibit great plasticity, with their anatomical location being diffuse. This is another feature that neuroscientists should be looking for.

Though, it may not be possible to reconstruct the evolutionary history of the causal factors for the brain systems that enable the formation of linguistic signs, we can nevertheless test whether such systems actually exist and whether they exhibit some of the predicted properties, such as plasticity and pleiotropy. There is already evidence in support of the hypothesis.

Concerning the existence of these offline systems, we can see them at work in language once we isolate their effects from those of other activities concurrent with language at the motoric and conceptual levels. For instance, Meister and Iacoboni (2007) report on an experiment in which they compare the processing of visual stimuli while performing an action perception task and two linguistic tasks. They did not find any area specifically activated or with higher activity during the two linguistic tasks: “when visual stimuli concerning object-oriented actions are processed perceptually, they activate a large bilateral fronto-parietal network. When the same stimuli are processed linguistically, they activate only a subset of this network and no additional areas” (p. 6). They argue that these results support “the evolutionary hypothesis that neural mechanisms for language in humans co-opted phylogenetically older fronto-parietal neurons concerned with action perception” (p. 6). The identification of neural systems involved in language, and their role, is extremely difficult. As Dehaene and Cohen (2007) point out, module sharing may involve all levels of brain hierarchic organizations: micro-maps (millimeter-size columns), meso-maps (centimeter-size circuits), and macro-maps (larger-size networks). But with the rapid progress in technology to probe the brain, scientists can refine the testing of linguistic properties relating to neural systems, and eventually put the hypothesis to a test.

Regarding plasticity, Hein and Knight (2008) provide evidence that the same brain region can support different cognitive operations (theory of mind, audiovisual integration, motion processing, speech processing, and face processing) depending on task-dependent network connections (see also Bookheimer, 2002, p. 153). There is no fixed macro-anatomical structure that is exclusively dedicated to language: linguistic processing is a widespread property of the neural networks (Fedor et al., 2009). Language exhibits extensive plasticity for the localization of its components between and within individuals (Neville and Bavelier, 1998), during its development (Karmiloff-Smith, 2006), in its repair (Hagoort, 2009), and depending on its modality (Neville, 1993; Mayberry, 2002). The often-noted association between human praxis and language also points in the same direction. There is a genetic linkage between handedness and language dominance, and clinical correlations between aphasia and apraxia (Donald, 1998).

Regarding pleiotropy, if human brains have systems of neurons that are functionally less specialized, systems that can be activated *in absentia*, triggered by representations of events instead of the events themselves, and produce representations of events with no brain-external realization, then we should find evidence for this capacity in other functional traits unique to humans. There is compelling evidence that several interrelated traits are uniquely human, and absent or in very rudimentary forms in other animals (e.g., Premack, 2004; Penn et al., 2008; Fedor et al., 2009). This Human-specific Adaptive Suite extends across many domains and involves qualitatively huge differences from species that are closely related to us. Here is an indicative list, with a few of the relevant references.

Human-specific cognitive traits

1. Language: signs and syntactic combinations
2. Imitation (Meltzoff and Moore, 1997; Rizzolatti and Craighero, 2004; Karmiloff-Smith, 2006)
3. Advanced Theory of Mind (Flavell, 1992; Povinelli, 2000)
4. Detachment from immediate situation, episodic memory (of noncurrent scenes and events) (Gärdenfors and Osvath, 2005)
5. Object permanence (Hurford, 2007b)

Human-specific neurological traits

6. Brain with large amount of neurons and increased connectivity (Russo and Treves, 2011, Deacon, unpublished)
7. ApoE4 (apolipoprotein E4) (provides better synaptic interactions) (Bufill and Carbonell, 2004) and other proteins with effects on language (Fitch et al., 2010)
8. Plasticity of the brain for several functions (Fedor et al., 2009; Hagoort, 2009)
9. Offline Brain Systems (offline activations, inhibiting input or output) (Hurley, 2008)

The human-specific cognitive and neurological traits are so closely linked that several scholars assume that at least a good part of them coevolved synergistically from a common factor underlying these various cognitive modules (Szathmáry, 2008; Fedor et al., 2009). Some assume that the underlying supermodule is one of the functional modules, the two most popular being Theory of Mind and language. However, Penn et al. (2008) argue compellingly that the suite of discontinuities between human and non-human minds cannot be explained by relating an *explanans* directly to the functioning of these cognitive domains. (See Bouchard, 2013, pp. 113–114) for arguments against the language-first and ToM-first hypotheses). The Human-specific Adaptive Suite provides initial evidence for a neurobiological innovation with general representational potential. Given the limitations in the current techniques available, the specifics of many of these traits are still unclear, but they may ultimately help us resolve the problem of the neural basis of sign formation.

Though this is difficult, the hypothesis can nevertheless be tested. One useful line of inquiry can be found in recent experiments by Stanislas Dehaene and Laurent Cohen (Dehaene, 2005; Dehaene and Cohen, 2007). They show that some adaptations can occur much faster than is expected on a genetic scale, due to a process that they call “neuronal recycling” that operates during cultural acquisitions such as reading and arithmetic. They observe that part of the human cortex is specialized for these two cultural domains. Since invention of these cultural activities is too recent to have influenced the evolution of our species, they hypothesize that this specialization results from neuronal recycling: reading and writing are not genetically encoded, but they nevertheless find their niche in a well-suited set of neural circuits.

Note that under the hypothesis that the novel brain systems coincidentally allowed mental states corresponding to elements of the perceptual and conceptual substances to meet in our brains to form linguistic signs, this does not raise what Chomsky (2005, 2007, 2010, 2011) refers to as the Jacob-Luria problem. Though he accepts that pressures to communicate may have played a role in the gradual fine-tuning of language, Chomsky has repeatedly claimed that, at its origin, language could not have evolved due to communicative pressures because this raises a problem:

(7) *Luria/Jacob problem*: How can a mutation that brings about a better communication system provide any survival advantage to the first single individual who gets it?

A mutation occurs in a single individual, whereas communication takes place between individuals^6^. Under my hypothesis, the offline systems with general representational capacity took on this other function of linking percepts and concepts after they were in place due to a suite of evolutionary pressures. The Luria/Jacob problem does not arise in this approach because the change was not for language or any of its functions like communication or organizing thought. The change produced offline systems. Linguistic signs are a side effect of this neurobiological property. Even if it depended on a mutation (but I doubt this to be the case as indicated above), the new trait could spread in a population because it has evolvability of its own, and all the members of that group are then brain-ready for the innovative side effect when it occurs: by the time words come around, they can be understood by conspecifics.

The advent of some kind of DR system is the crucial small change that made a big difference. This provided the core biological mechanism of the language phenotype—the capacity to link percepts and concepts into signs. Given this capacity and the prior properties of the two substances of the elements linked by signs, the rest of the linguistic properties follow without the need of any additional language-specific rules or conditions. In the next sections, I show how this happens in the three core components of grammar: phonology, semantics, and syntax.

Before turning to these issues, an important question remains to be addressed. Once humans had the capacity to form a limitless number of signs, they developed a capacity to learn and remember a vast set of such signs. How exactly this additional capacity depends on the mechanisms of the first capacity is a question that can now be asked, given my hypothesis. If I am correct in supposing that the DR system is likely to be (part of) what provides humans with a more advanced Theory of Mind (such as a shared attention mechanism and a meta-representation of others' mental states, Baron-Cohen, 1995), and if it also turns out to be correct that word learning strongly depends on an advanced ToM (Bloom, 2000), then the DR system would be crucial for both the capacity to form signs and the capacity to learn and remember them. See the discussion in section 9.5 of Bouchard (2013) and references therein.

## Contrastive dispersion of percepts and combinatorial phonology

As is the case in other biological systems, DR systems are complemented by epigenetic self-organizing constraints that emerge from interactions among properties of building materials that limit adaptive scope and channel evolutionary patterns (Jacob, 1982; Erwin, 2003). Since the linguistic linking between a percept and a concept is arbitrary—that is, it is not hard-wired but made possible by their representations in DR systems—the representation of any percept can potentially be linked to the representation of any concept, and the links can change very rapidly. So there are innumerable possible links. This is compounded by the fact that there are infinitely many incrementally different vocal forms that we can produce and perceive, and an untold number of possible concepts/signifieds because DR systems introduce a detachment from the immediate situation that opens the door to any imaginable situation, presented from a multitude of perspectives. Moreover, there is the logical possibility that individuals will choose different linkings: in the extreme case, each individual would have its own system. Therefore, DR systems introduce an unprecedented sort of chaotic system in the brain. This creates randomness that is confronted with material constraints. As in other situations far from equilibrium, small chance disturbances are progressively amplified by material properties and result in clusterings, in order out of chaos (Prigogine and Stengers, 1984).

In this kind of self-organization, local interactions of components of a system generate complex organized structures on a global scale. In language, the potential chaotic dispersions of arbitrary signs are constrained by the physical and cognitive properties with which the signs are confronted. These constraints restrict the linguistic sign system in a way that maximizes contrastive dispersion and creates clusterings that result in the various properties of language that we observe in phonology, semantics, and morphosyntax.

### Phonological segments

The signifier/percept of a sign is the part most noticeably influenced by material properties. Though the representation of any percept at all could in theory become a signifier, the possibilities of the chaotic system are considerably narrowed by material properties of our production systems and perception systems.

A salient property of human vocalizations is that they are perceived as segments: discrete elements. This is a general design feature of human neurophysiology: information that unfolds over time is chunked in the acoustic domain, as well as in other domains such as vision. This is a bilateral stimulus-neutral system of temporal segmentation that operates before feeding specialized lateralized systems such as the processing of speech or music (Poeppel, 2001). Sensory input is analyzed on different timescales by the two hemispheres. High-pass (global) information from short 20- to 50-ms temporal integration windows is passed to left hemisphere areas, whereas low-pass (local) information from long 150- to 250-ms integration windows is passed to the right hemisphere (Poeppel, 2001) (However, the issue is still unclear and recent work shows that lateralization in this case may be weak; Giraud and Poeppel, 2012). These oscillations arise naturally in our perception of vocalizations (Poeppel, 2003; Sanders and Poeppel, 2007), and the temporal integration of vocalizations is reflected as oscillatory neuronal activity. The timings correspond to typical segments and syllables.

Similar, bilateral segmentation systems appear to be shared by other species; they are the basis of the auditory processing of species-specific vocalizations in macaque monkeys, and the ability of squirrel monkeys to discriminate between conspecific and non-conspecific vocalizations (according to studies reported in Poeppel, 2001). This timing ability is the basis of a system with an important adaptive benefit: a strong change in rhythm signals danger. In sum, we perceive sound as segments, in a digital, not analog way. Segments are perceived as being produced concatenated. An important question is what determines the particular repertoire of possible phonemes. Why do digitized vocal percepts cluster in a few particular hot spots among the innumerable, chaotic possibilities we can produce and perceive? As in other chaotic systems, the clusterings depend on frequency and accumulation: chance vocalizations are progressively amplified by material properties pertaining to ease of production and distinctness of perception. On the production side, vocalizations involve the displacement of organs, hence muscular energy. Certain vocalizations are easier to pronounce and require less energy; this is likely to favor their use and increase their frequency (Lindblom, 1992).

The human perceptual systems also set upper bounds on the distinctions that we can perceive or produce as signifiers. Distinctness of expression is particularly important in the case of acoustic information since it is only physically available for a very short length of time and cannot be recovered in the case of an erroneous perception. Nowak et al. (2002) found that the demands of discriminability (as well as memory and time to learn) constrain the system to a fairly small set of signals, an observation already made by Wang (1976, p. 61). The actual repertoire is very small: a few dozen discrete perceptual elements. This observation extends to sign languages that use the gestural modality: there are very few gestural minimal elements, and like phonemes, they are made up of articulatory features (see, for instance, Brentari, 2002). This small set of percepts is a result of self-organization. Vocalizations that are easier to produce and can be more distinctly perceived have a higher frequency of use. As frequencies increase, accumulations occur at certain points in the articulatory–acoustic continuum. Percepts cluster in particular hot spots as a result of this contrastive dispersion. As Lindblom (1992) (following Liljencrants and Lindblom, 1972) indicates, a compromise between perceptual distinctiveness and articulatory cost brings about quasi-optimal perceptual distinctiveness. But this is not sufficient, because the search space is too large for convergence on a structure as complex as the human phonological system. However, if we take into account the properties of building materials, self-organization derives the phonemic clusters. Thus, Carré and Mrayati (1990) and Oudeyer (2005, 2006, 2007) show that canalization by the vocal tract and general acoustic theory define “eight discrete regions of such a tube where deformations, or constrictions, afford greatest acoustic contrast for least articulator effort” (Studdert-Kennedy, 2005, p. 64), and these correspond to places of articulation in natural languages. Thus, vocalic systems most frequently have peripheral vowels, which are the most contrasted (Ménard, 2013).

### Phonological combinations

This severe limitation on the number of usable percepts is the source of the clash between the possibilities of the perceptual and conceptual substances. There are innumerable meanings and ways to partition meaning (more on this below), but discriminable speech sounds are limited by the material properties of sound production and perception. The combinatorial formation of signifiers is usually attributed to this clash between the possibilities of the two systems. “If the symbols were holistic vocalizations like primate calls, even a thousand symbols would be impossible to keep distinct in perception and memory” (Jackendoff, 2002, p. 242). In simulations like Oudeyer's, the small number of clusters “automatically brings it about that targets are systematically re-used to build the complex sounds that agents produce: their vocalizations are now compositional” (Oudeyer, 2005, p. 444). How could that be? Where do the compositional processes come from? The answer is again found in the material properties already present in the forms. Vocal units have the following universal material properties:

_ they occur in time, so they can be ordered and juxtaposed;
_ they can have various intonations;
_ they can be shortened or lengthened;
_ they can be stressed or unstressed.

These acoustic and auditory properties are also distinguishing elements in the signals of other mammals (Lieberman, 1968).

Vocalizations occur in time, and the material properties of vocal articulators are such that we cannot produce more than one vocal unit at a time. This is a contingent property of language production. Since vocal units are aligned in time, our perceptual system captures the linear properties of vocalizations when they are produced, in particular the linear relationship between two vocal units, the most salient one being linear adjacency. The linear adjacency of two vocal percepts is itself a percept and can be represented by a DR system, like any other percept. The relational percept of juxtaposition is already in the stock of our perceptual system; hence, it is available for DR systems that link concepts and percepts. Another material property of vocalizations is intonation; therefore, another perceptual element represented by DR systems is the tone superimposed on a vocal unit, of which there are a few distinctive values due to contrastive dispersion. Similarly, the length and stress of a segment are percepts that can be represented by DR systems, within the limits of distinctive values. Crucially, in an arbitrary system, the percept represented by a DR system and linked to a concept can be any element among those recognized by the perceptual system: a vocal unit, a juxtaposition of units, an intonation, a length, or a stress. Because the system is arbitrary, it makes no difference whether the represented element is simple or complex. The acoustic image can be a single phoneme or the relational percept of juxtaposition applying any number of times to phonemes, as well as any of the available distinctive intonations, lengths, and stresses on these elements. These complex elements remain within the limits of what humans can distinctively perceive or produce because their parts have the appropriate qualities. Phonological combinatoriality comes from a material property of the articulatory and perceptual systems, namely the fact that vocalizations are temporally linearized, which entails the percept of juxtaposition. The phonetic data provide information on the source of more abstract principles: segmenting into phonemes, as well into as words/morphemes, already contains computational properties (see DeWitt and Rauschecker, 2011 for combinatorial properties in basic perception). This simple concatenation-recursion of phonemes allows an unlimited derivation of signifiers: any combination of distinguishable percepts can be a signifier. This system is subject to a general law of nature whereby the frequency of an element is inversely correlated with its complexity: the simpler an element is, the more likely it is to appear in nature (cf. Zipf, 1965/1949). Though concatenation-recursion of phonemes can derive infinitely complex signifiers, the simpler ones are much more likely to be formed, produced, or heard. This higher frequency creates accumulations that make the system relatively conservative in terms of the number and complexity of elements that form its signifiers. In addition, production ease and auditory salience influence not only the dispersion of vowels and consonants, but also syllabic templates, or sequences of segments: the combinations of phonemes are subject to phonotactic constraints, such as the energy expended for the transition, which also constrain the nature and number of potential signifiers. The constraints that arise from properties of the articulators and ease of articulation influence what phonemes occur in adjacent positions as early as babbling (MacNeilage and Davis, 2000). The overall complexity of a signifier is also likely to be limited by memory and retrieval capacities.

Discrete speech sounds and their combinations emerged because they are consequences of material laws that apply to a certain kind of organism hosting DR systems that can represent elements of their perceptual and conceptual systems and links between them. The chaotic system deriving from these brain systems must have the properties that we observe because the building materials channel the way the system becomes structured into specific self-organizations.

## Contrastive dispersion of meanings and combinatorial semantics

Segmentation is also a design feature of the human cognitive make-up. We digitize the world and events into discrete chunks, action packages varying from 0.3 to 12 s, mostly 1 to 4 s long (Schleidt and Kien, 1997). As for the ontology of the cognitive units, our perceptual attention systems treat the world as containing two basic kinds of entities (Hurford, 2007a, p. 527):

1. objects (“something is there”);
2. properties of these objects (“what is there” “what is happening to it”).

Another aspect of cognitive segmentation is found in the two types of attention discussed by Humphreys (1998). Global attention captures the gist of the whole scene. In language, this corresponds to something like the main predicate and its arguments. Local attention is subsequent focal attention on local features of individual objects. In language, this corresponds to secondary predicates such as nouns, adjectives, etc.

By allowing detachment, DR systems introduce a chaotic expansion on the meaning side of language: there is an extremely large if not infinite number of potential (offline) concepts. First, the vast number of objects and situations we perceive can all be represented offline as concepts, as well as their properties. This is compounded by the various perspectives we can have on them (Quine, 1960). Moreover, the potential for concept formation is multiplied by the affordance of intra-brain interactions where some neuronal systems are triggered by other brain events. In addition, a particular language can partition the conceptual substance in countless possible ways to delimit its lexical meanings (Saussure's radical arbitrariness). In a system of arbitrary signs, any of these elements treated by the cognitive system could be a meaning represented by DR systems and could be linked to a vocal form.

But this unbridled expansion in meanings is constrained by design features of our cognition. For instance, our global attention process is constrained as to the number of participants that it can take in at a glance: we can subitize at most four salient objects at a time (cf. the “magical number 4” in Hurford, 2007b). Though actual events can involve any number of actants, the chaos of what we observe is organized by subitizing and chunks of four or fewer actants. The recurrence of the perception of these chunks in the environment creates accumulations, and language has settled on predicates with at most four arguments. The chaotic expansion that could potentially arise from linguistic arbitrariness in the meanings of words is also limited in a more general way by material properties and self-organization. Here, too, order arises out of chaos and clusters are formed in the mass of the conceptual substance as a result of frequency and accumulation. In this case, accumulation depends on the material conditions that make the situations denoted by the concept relevant for the organisms. The more a situation has some importance and/or is encountered frequently by the organism, the more frequently concepts associated with it will be activated. The accumulations self-organize around the concepts most used by the organism. It is this usefulness that makes the meanings tend to correspond to fairly broad and/or usual categories of things, actions, qualities, etc. (an observation already found in Locke, 1690/1964, p. 15). Similarly, Nowak et al. (2002, p. 2131) note that “[t]he evolutionary optimum is achieved by using only a small number of signals to describe a few valuable concepts.”

Usefulness is also the motivation for the important role played by basic level concepts (Rosch and Mervis, 1975; Rosch et al., 1976). Murphy and Lassaline (1997) argue that the basic level is an optimal compromise between informativeness and distinctiveness: this level is informative, because we can infer many unobserved properties once we know which basic category something belongs to, and distinctive because it is a relatively easy categorization to make. Thus, if you ask someone What are you sitting on?, you are more likely to get the answer chair rather than a subordinate such as kitchen chair or a superordinate such as furniture. Names for basic-level concepts are among the first common nouns learned by children (Brown, 1958).

In fact, we can construct so many particular objects and events and their properties out of reality, potentially an infinite number, that it would not be useful (in a general as well as in an evolutionary sense) since most of them recur only very rarely, if at all. This is likely why meanings tend to converge on these hot spots of accumulation.

Even with an important number of U-signs and the possibility of combining them by means of C-signs, the resulting meanings are nevertheless generally quite broad and may correspond to several different situations in the world, including the meanings of sentences. Trying to remedy this underdetermination would force language into ever more complex constructions, to a point where it would be extremely unwieldy. Humans have another prior mental attribute that avoids this problem and favors the cumulative use of broad concepts: a system of pragmatic inferences that derives from a full Theory of Mind (ToM). Given the pragmatic inferences that derive from ToM and the context of utterances, expressions need not have fully determined meanings in order to convey information that is sufficiently precise to be of current use. When two human beings interact, they each have a full ToM, similar cognitive and perceptual systems, and similar contextual information. Therefore, they both know that they have an enormous amount of information in common, and their language faculty does not operate in a vacuum. Using and understanding language involves intensive reliance by speakers on their shared conceptual and contextual knowledge. Pragmatic theories from authors as diverse as Ducrot (1984), Grice (1975), Levinson (2000), and Sperber and Wilson (1986) all share this observation that comprehension is inferential and it draws on both sentence meaning and context (in a very broad sense). Since the inferential system is independently grounded, languages do not drift into an unbridled multiplication of meanings redundant with contextual information, but converge on broad, sufficiently informative meanings (Bouchard, 1995; Hoefler, 2009). A similar argument can be made from the perspective of language's other main function, i.e., thought organization.

To sum up, discrete meanings are clusters formed in the mass of the conceptual substance as a result of maximizing contrastive dispersion across the space for signifieds under the effects of frequency and accumulation due to relevance/usefulness. These clusters are relatively few in number and signs tend to have fairly broad meanings. This does not adversely affect the communicative or thinking functions of language because linguistic signs reside in organisms that independently have an inferential system that supplies the required complementary information.

## Syntax

### The source of syntax

Syntactic combination of words and phrases raises the same question as phonological concatenation. Where do the combinatorial tools come from?

If we try to determine what brain systems enable the formal properties of syntactic combinations and the plausibility of these systems given known laws of evolution, it is likely that we will not get very far, because formal systems are only very remotely related to factors involved in evolutionary changes. The system that forms signs (lexicon) and the system that combines signs (syntax) have properties that are so different in current models that they seem quite disconnected. For instance, Chomsky (1995, p. 8) says that matters concerning “the sound–meaning pairing for the substantive part of the lexicon […] appear to be of limited relevance to the computational properties of language.” But that is not so in the approach I adopt. If we look at the physiological and cognitive properties of the elements being combined, a hypothesis emerges with means and a method of confirmation that are clear enough to be verifiable. Since I argue that the syntax of a language is a set of particular combinatorial signs, each with its signified and signifier, I change the ontology of syntax from a formal computational system to a set of neurophysiological elements.

Syntactic compositional processes, i.e., C-signs, are simply functional uses of universal pre-existing properties of vocal sounds and universal pre-existing properties of our cognitive system. Combinatorial syntax is due to the self-organization of these prior vocal and cognitive elements. On the conceptual side, the most frequently represented element is the relation of predication, since it is common to all the attributions of properties. This is compounded by the fact that human brains with DR systems have extended this cognitive process: DR systems can not only attribute properties from sensory inputs to perceived objects but, by operating offline, they can also attribute abstract conceptual properties, not linked to immediate sensory inputs. Predication is the broad meaning par excellence. It is a relation that is broad enough to apply to almost all possible meanings and it is omnipresent in our cognitive system. So it is the meaning that creates by far the strongest concentration point in the chaos of semantic DR systems. The fact that our linguistic system has integrated the predicative function at its core simply reflects the place of this readily exaptable concept in our cognitive system, its high rate of frequency and accumulation.

On the perceptual-physical side, words being made of concatenated phonemes, i.e., of elements with properties of vocal sounds, the most frequent elements are temporal sequencing, and superimposition such as intonation, stress, and length. These traits are always present, so they are by far the most frequent elements in the vocal perceptual system. Thus, the hottest accumulation point in the mass of the conceptual substance is the relational concept of predication, and the hottest accumulation points in the mass of the perceptual substance are the two relational percepts of juxtaposition and superimposition. These accumulation points are so overwhelmingly dominant in their respective domains that they increase the frequency of links involving them to the point where these links inevitably accumulate and crystallize. It follows that when human organisms develop signs due to properties of their prior DR systems, they inescapably develop combinatorial signs involving predication as a meaning and juxtaposition and/or one of the forms of superimposition as a signifier. In short, syntax is a consequence of self-organization arising out of the chaos created by DR systems, as is the linguistic sign.

Syntactic combination arises from prior properties of the conceptual and perceptual substances involved, given general laws of nature concerning highly complex systems, à la Prigogine and Zipf. These cognitive and material design properties have a very strong canalizing effect. In particular, they are all primitive combinatorial processes: predication combines an object and its property; order and juxtaposition hold of two segments; intonation, length, and stress apply to segments. As a result, the sign itself introduces combinatorial systems into the linguistic system, and from these primitive combinatorial systems derive concatenation in phonology and combination in syntax. The logically prior properties of the physical and conceptual components of signs are the source of key design features of language, including the particular type of combinatorial system that it has. Syntax happens to have functional effects that are useful for communication and thought, but they are not the factors that triggered its emergence; they are just fortunate consequences.

### Type-recursion

In addition to concatenation-recursion, as found in phonology, the syntax of human language exhibits a particular kind of recursion, where an element of type X can be embedded within other X elements indefinitely. I refer to this as type-recursion. We want to know not only why language has recursion, but also why it has type-recursion.

Type-recursion involves more than recognizing nested attributes of objects (an ability that some animals have) (Penn et al., 2008, p. 117). To have type-recursion, you need an additional property: the complex signs must have a label; they must belong to a category. If a phrase did not have a labeled category, it could not contain another phrase of the same category.

Since properties of signifiers are essentially those of phonological elements, the types cannot come from these. The source of the typological distinction must be in the signified/meaning. Whether these categories are determined ontologically or functionally is an important question that has been debated for centuries. I will not address it here since it is tangential to the issue. I will simply assume the broad hypothesis that lexical items have categories when they interact in syntax. It is also broadly assumed that the phrasal categories are identical to the lexical categories (Noun, Verb, Adjective, Preposition, Tense, etc.). This is due to the fact that syntactic phrases are endocentric: the category of a phrase always comes from one distinctive component, which we refer to as the head—ultimately a lexical head, a U-sign.

The syntactic properties of headedness and endocentricity derive from prior properties. First, asymmetries in syntactic relations, such as the asymmetry between heads and dependents, come from the fact that predication, the meaning of C-signs, is asymmetrical (Venneman, 1974; Keenan, 1978; Bouchard, 2002): the property expressed by the dependent is attributed to the head. Second, endocentricity derives from the way we cognitively process property-attribution (predication): in our cognitive perception of the world, an object to which we attribute a property remains an element of the same type; in a way, it remains the same object. In language, this means that a noun to which we add an adjective remains a nominal thing; a verb to which we add an argument remains a verbal thing, etc. There is a kind of hyperonymic relation between the head and the phrase (cf. Bauer, 1990; Croft, 1996). Assuming the parsimonious hypothesis that the only syntactic primitives are lexical and combinatorial signs, we derive the Endocentricity Theorem:

(8) Endocentricity Theorem

The category of a constituent X is the category of the element that receives a property by the predication of the C-sign that formed X.

In other words, if X is formed by a C-sign that assigns the property of A to B, then X's category is the category of B (B being the “object” that receives the property of A).

We now see why language has type-recursion: type-recursion occurs whenever a restraining sign or one of its elements happens to be of the same type as the restrained sign whose category projects and determines the category of the complex sign. Type-recursion is a side effect of the combinatorial properties of the substances of signs, interacting with a general cognitive principle of property attribution.

Combinatorial syntax is not a hard-wired property that has evolved at some time. This ability ultimately derives from the particular representational capacity of DR systems that allows the formation of signs. Discrete infinity is a side effect of limitations on chaotic systems like arbitrary language, in interaction with material properties of the sensory-motor and conceptual substances. Both concatenation-recursion and type-recursion derive from the resulting self-organization that takes place. The reason that other animals do not have anything like combinatorial syntax in their communication systems is that they do not have DR systems, which also explains why they do not have unit-signs/words. The crucial leap for a language-ready brain was the development of DR systems that enable the linking of elements of two substances with no logical or natural connection between their elements, so that the linking is purely symbolic. The sole distinctive trait of human language is the capacity to form Saussurean signs. Recursion and discrete infinity are just side effects of this trait.

The ontology of syntax is not a formal computational system, but a set of neurophysiological elements. These elements have high evolvability, in contrast with formal systems. Interestingly, it is by attributing a non-central place to recursion that we can explain how language became type-recursive. My account is in the spirit of Evo-Devo proposals: type-recursion is not due to a specific genetic change but to logically prior properties of the building materials of language.

The Sign Theory of Language has high evolvability with respect to signs and combinatoriality. But of course, a linguistic theory must also pass the test of accounting for the collection of properties that linguists have uncovered about language. I am fully aware that if I am to make the radical claim that syntax is just a small set of C-signs determined by the nature of the sensory-motor and conceptual substances, I must show how that proposal can account for the numerous claims made about the syntax of human languages over the years. Space limitations prevent me from doing that here. But the linguistically inclined reader will find a long discussion in Part IV of Bouchard (2013) that tackles a representative sample of some of the constructions that have been most influential for theoretical argumentation over several decades:

- subject-auxiliary inversion and structure dependence;
- binding conditions on referential relations;
- existential *there*-constructions;
- subject raising constructions;
- long distance dependencies and bounding conditions.

In addition, Bouchard (2002) analyses the distribution and interpretation of adjectives in French and English in exquisite detail, as well as bare noun phrases and bare determiners (clitics).

In all these cases, the unification proposed in STL leads to new insights that allow us to progress in our understanding of language. Many properties that we know about make sense in this model, whereas they just existed, were described but left unaccounted for, in classic models.

## Conclusion

We must understand precisely what language is and have well defined linguistic phenotypes to search for the neural substrates that enable these phenotypes.

From a linguistic perspective, there are strong reasons to assume that the central trait of human language is the capacity to form signs by linking perceptual forms and meanings, rather than the currently prominent view that puts computational tools with discrete infinity at the core of language. Recursive syntax turns out to be a side effect of sign formation: due to general principles, an organism that develops signs inevitably develops combinatorial signs. The Sign Theory of Language offers a comprehensive and unifying approach to the functioning of the main subsystems of language: by calling on the perceptual and conceptual substances of signs and the self-organization that it triggers, the theory explains specific properties of signs, as well as the basic structuring of language in its phonology, semantics, and syntax.

This change of perspective regarding linguistic phenotypes suggests to direct research on neural substrates that enable meta-representational functionalities, detached from sensory input and motoric output. Such Detached Representational systems need not be language specific. Given biological continuity, it is likely that the neural mechanisms will have broad effects at the functional level. While looking for the neural substrates that enable the formation of linguistic signs, it may therefore be useful to consider possible effects of these substrates on non-linguistic traits that may also depend on DR systems, such as the traits discussed under the Human-specific Adaptive Suite.

Another important question in probing the language-ready brain in adults is how much of the mechanisms are in place at birth and how much of the language system takes form during infant development.

My hope is that the change in perspective that we are led to from purely linguistic considerations will enlighten the search for the neural substrates of language, so we will eventually have a better understanding of what makes, and made, the human brain language-ready.

The general outline of the model is as follows:

(9) Figure 3

**Figure 3:**
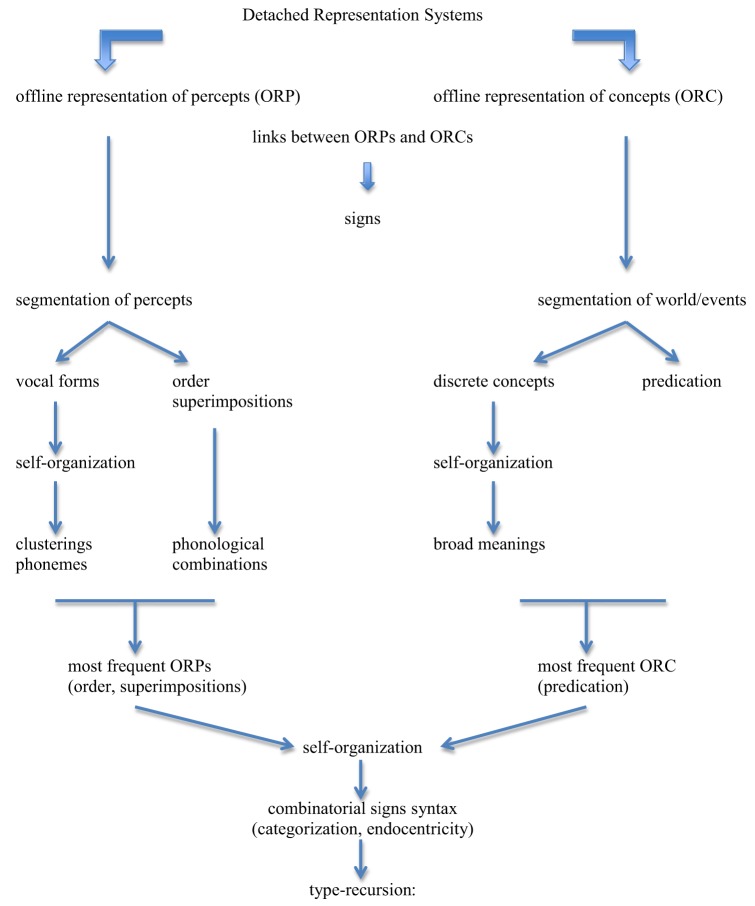
**General outline of the model**. Detached Representation Systems produce offline representations of percepts (ORP) and offline representations of concepts (ORC). Links between ORPs and ORCs create linguistic signs. These signs have restricted traits because the prior properties of the substances of percepts and concepts severely constrain them. The segmentation of percepts produces a potentially infinite set of vocal forms; these forms undergo self-organization that creates clusterings that result from frequency and accumulation due to ease of production and distinctness of perception: this delimits the set of potential phonemes for languages. The segmentation of percepts also introduces order and superimposition (intonation, length, stress), which derive phonological combinations. The segmentation of world/events produces a potentially infinite set of discrete concepts; these concepts undergo self-organization that creates clusterings that result from frequency and accumulation due to relevance/usefulness for the organism: this delimits the set of broad meanings for languages. Concepts fall in two broad classes, objects and properties, the latter introducing the notion of predication in a broad sense. The most frequent ORP elements are order and superimposition, and the most frequent ORC element is predication. These accumulation points are so overwhelmingly dominant in their respective domains that they increase the frequency of links involving them to the point where these links inevitably develop into combinatorial signs (syntax). Given categorization and endocentricity (due to object permanence), the syntax of languages has the formal property of type-recursion.

### Conflict of interest statement

The author declares that the research was conducted in the absence of any commercial or financial relationships that could be construed as a potential conflict of interest.

## Footnotes

^1^This paper presents the main hypotheses exposed in Bouchard (2013). I have therefore borrowed substantially from that text without indicating it by quotation marks or references in order not to overburden the readers.

^2^For instance, here is an illustrative sample of UG elements taken mostly from Hornstein and Boeckx (2009) and Narita and Fujita (2010):

- endocentricity labeling;
- c-command;
- uninterpretable features and specifications about which elements they may attach to and when;
- numerous functional categories;
- an intractable number of micro or macro parameters distributed over different modules;
- binding conditions for pronouns;
- displacement;
- agreement;
- constituency;
- cycle/phase bounding nodes;
- Phase Impenetrability;
- Transfer;
- locality conditions (Ross's Problem: why locality holds for Move but not pronominalization);
- condition on theta assignment: arguments must be initially merged in theta-positions;
- Linearize: there has to be a procedure Linearize, with something like the Linear Correspondence Axiom (Kayne, 1994) to constrain it.

^3^Moreover, the UG problem has actually increased, since analyses in that model have drifted toward a constant increase in functional categories (to wit, the cartography approach, Cinque, 1994, 1999, 2002; Belletti, 2004; Rizzi, 2004 and nano-syntax Kayne, 2010). Most of these functional categories are redundant system-internal correlates (there are functional categories of SIZE, COLOR, ORIGIN, etc. because there are adjectives of those categories): they add nothing to our understanding of the facts. They are not even discovered correlations but invented correlations, elements added to the theory solely to correspond to some phenomenon (much in the behaviorist way so fiercely criticized in Chomsky's, 1959 review of Skinner; see the discussion in Bouchard, 2001).

^4^In this paper, I compare my view with that of Chomsky, since it is the most influential one. There are many other theories of language and its origin, some of which relate to the brain and machinery prior to language. Because of space limitations, I cannot do them justice here, so I refer the interested readers to the extended discussion of other approaches in Bouchard (2013).

^5^In addition to these very direct ways of indicating that there is a relation between two signs, we can also indicate that a relation is being established between two percepts by physically shaping one in a conventionalized way, in a paradigm that indicates what relation is being established with the other, as with Case marking or agreement.

^6^Pinker (1994: 365), foreseeing the objection, counters that the initial grammar mutant could talk to the 50% of brothers and sisters, and sons and daughters, who shared the new gene.
